# Crystal structure of *O*-isopropyl [bis­(tri­methyl­sil­yl)amino](*tert*-butyl­amino)­phosphino­thio­ate

**DOI:** 10.1107/S205698901402622X

**Published:** 2015-01-01

**Authors:** Oleksandr O. Kovalenko, Vasyl Kinzhybalo, Oleksii A. Brusylovets, Tadeusz Lis

**Affiliations:** a Department of Inorganic Chemistry, Taras Shevchenko National University of Kyiv, Volodymyrska 64, 01601 Kyiv, Ukraine; b Institute of Low Temperature and Structure Research, Okolna 2, 50-422 Wroclaw, Poland; c Faculty of Chemistry, University of Wroclaw, Joliot-Curie 14, 50-383 Wroclaw, Poland

**Keywords:** crystal structure, (tri­methyl­sil­yl)amino, phosphino­thio­ate, N—H⋯S hydrogen bonding

## Abstract

[Bis(tri­methyl­sil­yl)amino](*tert*-butyl­imino)­thio­phospho­rane reacts in benzene with isopropyl alcohol *via* 1,2-addition of an ^*i*^PrO–H bond across the P=N bond, resulting in the title compound, C_13_H_35_N_2_OPSSi_2_. In the mol­ecule, the P atom possesses a distorted tetra­hedral environment involving two N atoms from (Me_3_Si)_2_N– and ^*t*^BuNH– groups, one O atom from an ^*i*^PrO group and one S atom, therefore the mol­ecule has a stereocenter on the P atom but crystal symmetry leads to a racemate. In the crystal, a pair of enanti­omers form a centrosymmetric dimer *via* a pair of N—H⋯S hydrogen bonds.

## Related literature   

For details of the synthesis of [bis­(tri­methyl­sil­yl)amino](*tert*-butyl­imino)­thio­phospho­rane, see: Scherer & Kuhn (1974 ▸). For its chemical reactivity, see: Kovalenko *et al.* (2011*a* ▸,*b* ▸,*c* ▸, 2012 ▸); Rusanov *et al.* (1992 ▸); Scherer *et al.* (1978 ▸). For its applications in catalysis, see: Zhao *et al.* (2014*a* ▸,*b* ▸); Goldys & Dixon (2014 ▸); Samuel *et al.* (2014 ▸); Kawalec *et al.* (2012 ▸); Zhang *et al.* (2007 ▸).
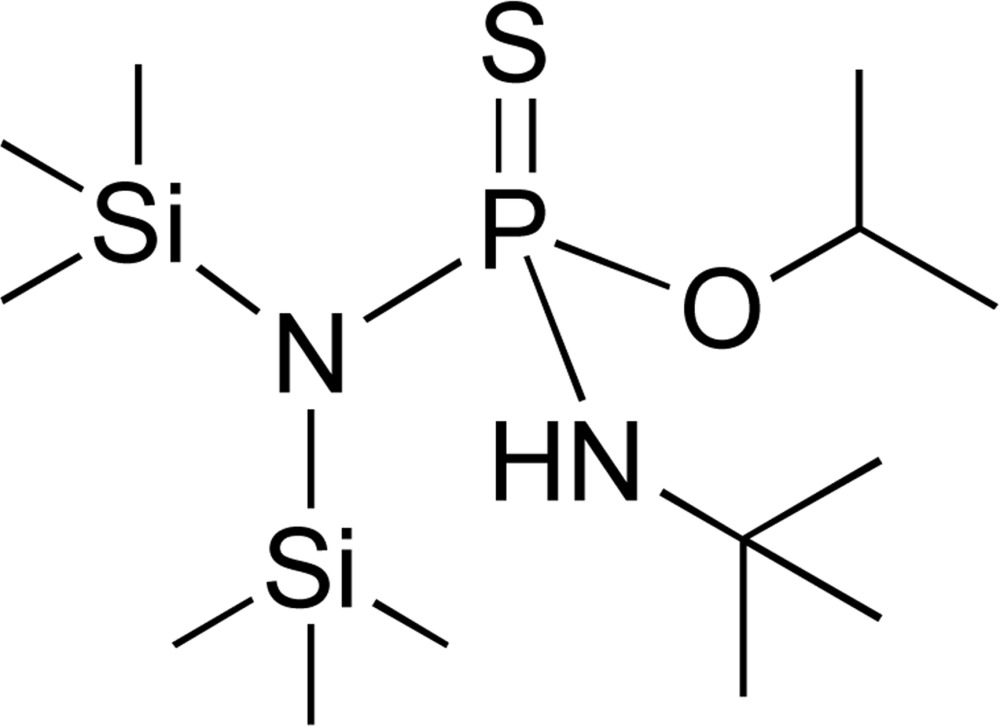


## Experimental   

### Crystal data  

C_13_H_35_N_2_OPSSi_2_
*M* *_r_* = 354.64
Monoclinic, 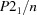

*a* = 9.942 (3) Å
*b* = 11.907 (3) Å
*c* = 17.726 (5) Å
β = 100.52 (3)°
*V* = 2063.1 (10) Å^3^
*Z* = 4
Mo *K*α radiation
μ = 0.35 mm^−1^
*T* = 100 K
0.30 × 0.20 × 0.20 mm

### Data collection  

Oxford Xcalibur PX κ-geometry diffractometer with a CCD area detector
36939 measured reflections
7436 independent reflections
5938 reflections with *I* > 2σ(*I*)
*R*_int_ = 0.033

### Refinement  

*R*[*F* ^2^ > 2σ(*F* ^2^)] = 0.041
*wR*(*F* ^2^) = 0.115
*S* = 1.08
7436 reflections
184 parameters
H atoms treated by a mixture of independent and constrained refinement
Δρ_max_ = 0.84 e Å^−3^
Δρ_min_ = −0.34 e Å^−3^

### 

Data collection: *CrysAlis CCD* (Oxford Diffraction, 2003 ▸); cell refinement: *CrysAlis RED* (Oxford Diffraction, 2003 ▸); data reduction: *CrysAlis RED*; program(s) used to solve structure: *SHELXS2014* (Sheldrick, 2008 ▸); program(s) used to refine structure: *SHELXL2014* (Sheldrick, 2008 ▸); molecular graphics: *ORTEP-3 for Windows* (Farrugia, 2012 ▸); software used to prepare material for publication: *publCIF* (Westrip, 2010 ▸).

## Supplementary Material

Crystal structure: contains datablock(s) I, New_Global_Publ_Block. DOI: 10.1107/S205698901402622X/xu5831sup1.cif

Structure factors: contains datablock(s) I. DOI: 10.1107/S205698901402622X/xu5831Isup2.hkl

Supporting information file. DOI: 10.1107/S205698901402622X/xu5831Isup3.cml

**Fig. 1.**
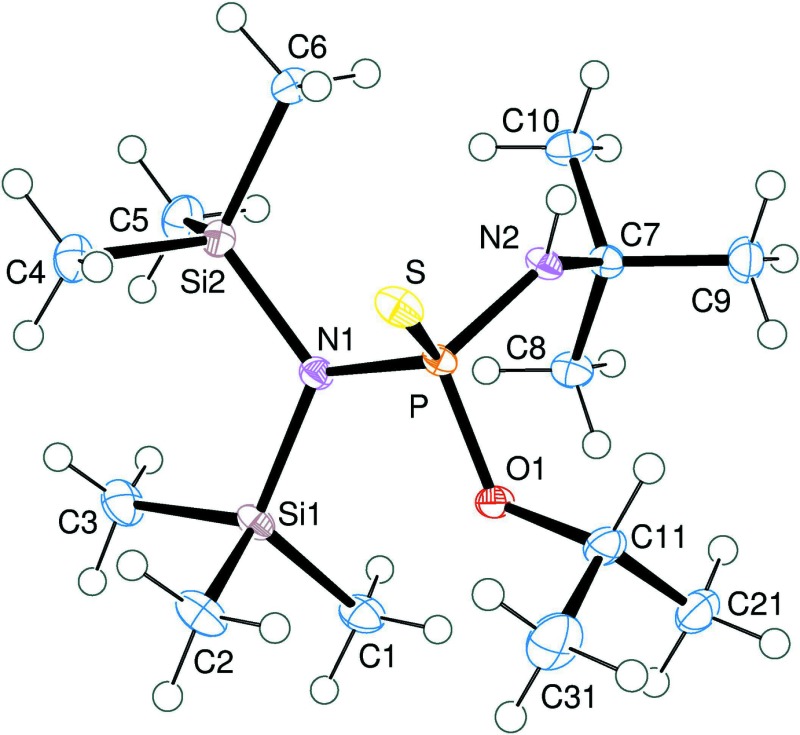
An ORTEP view of the molecular structure of the title compound, with atom labels and 50% probability displacement ellipsoids for non-H atoms.

ORTEP . DOI: 10.1107/S205698901402622X/xu5831fig1.tif
An *ORTEP* view of the mol­ecular structure of the title compound, with atom labels and 50% probability displacement ellipsoids for non-H atoms.

CCDC reference: 1036750

Additional supporting information:  crystallographic information; 3D view; checkCIF report

## Figures and Tables

**Table 1:** Hydrogen-bond geometry (, )

*D*H*A*	*D*H	H*A*	*D* *A*	*D*H*A*
N2H2S^i^	0.848(16)	2.631(16)	3.4326(13)	158.2(14)

Symmetry code: (i) 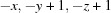.

## supplementary crystallographic information

### S1. Comments

[Bis(tri­methyl­silyl)amino](tert-butyl­imino)­thio­phospho­rane was first synthesized by Scherer and Kuhn in 1974, see: Scherer & Kuhn (1974), and later some general chemical reactivity of this compound was studied, see: Scherer *et al.* (1978). Based on these early results, penta­valent tricoordinated σ^3^λ^5^-phospho­ranes recommended themselves as promising ligands for the obtaining of new organometallic metallacycles with specific features. Recently we have reported and characterized series of transition metal metallacycles, containing phospho­rus atom in cyclic moiety, see: Kovalenko *et al.* (2011a, 2011b, 2011c, 2012); Rusanov *et al.* (1992). In current communication we reported the reactivity of [bis­(tri­methyl­silyl)amino](tert-butyl­imino)-thio­phospho­rane with iso­propyl alcohol. The reaction proceeds through a 1,2-addition of ^i^PrO–H bond across the P=N bond, resulting in the title compound. Resulted product was characterized by single X-ray analysis and ^1^H, ^13^C and ^31^P NMR spectroscopy. In these latter days it was discovered that low-coordinate phospho­rus compounds are catalytically active and might be efficiently applied in catalysis, see: Zhao *et al.* (2014a, 2014b); Goldys & Dixon (2014); Samuel *et al.* (2014); Kawalec *et al.* (2012); Zhang *et al.* (2007).

Central P atom posesses distorted tetra­hedral environment of four different substituents: (Me_3_Si)_2_N-, ^t^BuNH- and ^i^PrO- groups and S atom, resulting in stereocenter on phospho­rus. R and S isomers form centrosymmetric dimers due to the formation of a pair of N—H···S type hydrogen bonds. Geometrical parameters of *O*-iso­propyl [bis­(tri­methyl­silyl)amino](*tert*-butyl­amino)­phosphino­thio­ate are consistent with the values reported earlier (Rusanov *et al.*, 1992; Kovalenko *et al.*, 2011a, 2011b) for the compounds containing analogous phosphino­thio­ates, but deprotonated and coordinated to metal centers.

### S2. Experimental

All procedures were carried out under a dry argon atmosphere using standard Schlenk and glovebox techniques. Benzene and hexane were distilled from sodium-potassium alloy directly before use. Iso­propyl alcohol was dried and distilled from magnesium and stored over 4 Å molecular sieves prior to use.

In a Schlenk flask, (0.884 g, 3.0 mmol) of [bis­(tri­methyl­silyl)amino](*tert*-butyl­imino)­thio­phospho­rane was dissolved in 3 ml of benzene and the solution of iso­propyl alcohol (0.23 ml, 3.0 mmol) in 1 ml of benzene was added dropwise. The mixture was stirred for 1.5 h at room temperature, thereafter solvent was removed in *vacuo* producing an almost colorless tar. The residue was dissolved in 1 ml of hexane and kept at 252 K in order to induce further crystallization. Yield: 0.76 g, 71% of colorless crystals. ^1^H NMR (400 MHz, C_6_D_6,_ 298K): δ 5.00 (m, 1H), 2.45 (d, ^2^*J*_P—H_=10.3 Hz, 1H), 1.27 (d, ^3^*J*_H—H_=6.0 Hz, 3H), 1.19 (d, ^3^*J*_H—H_=6.0 Hz, 3H), 1.15 (s, 9H), 0.47 (18H); ^13^C{^1^H} NMR (100 MHz, C_6_D_6,_ 298K): δ 71.52 (d, ^2^*J*_P—C_=4.6 Hz), 52.72 (d, ^2^*J*_P—C_=4.6 Hz), 31.58, 31.53, 23.98, 23.92, 5.26 (d, ^3^*J*_P—C_=2.3 Hz); ^31^P{^1^H} NMR (162 MHz, C_6_D_6,_ 298K): δ 63.24 (dd, ^2^*J*_P—H_=10.3 Hz, ^3^*J*_P—H_=10.3 Hz).

### S3. Refinement

Positions of hydrogen atoms bonded to carbon were generated in idealized geometries using a riding model with *U*_iso_(H) = 1.5U_eq_(C) or 1.2*U*_eq_(C). The fractional coordinates of the H atom attached to N2 were identified from a difference Fourier map and refined freely with isotropic thermal displacement parameter.

### Crystal data

**Table:**

C_13_H_35_N_2_OPSSi_2_	*F*(000) = 776
*M**_r_* = 354.64	*D*_x_ = 1.142 Mg m^−^^3^
Monoclinic, *P*2_1_/*n*	Mo *K*α radiation, λ = 0.71073 Å
*a* = 9.942 (3) Å	Cell parameters from 25646 reflections
*b* = 11.907 (3) Å	θ = 4–32.6°
*c* = 17.726 (5) Å	µ = 0.35 mm^−^^1^
β = 100.52 (3)°	*T* = 100 K
*V* = 2063.1 (10) Å^3^	Block, colourless
*Z* = 4	0.30 × 0.20 × 0.20 mm

### Data collection

**Table:**

Oxford Xcalibur PX κ-geometry diffractometer with a CCD area detector	5938 reflections with *I* > 2σ(*I*)
Radiation source: fine-focus sealed tube	*R*_int_ = 0.033
Graphite monochromator	θ_max_ = 32.6°, θ_min_ = 4.7°
ω and φ scans	*h* = −15→14
36939 measured reflections	*k* = −17→18
7436 independent reflections	*l* = −26→26

### Refinement

**Table:**

Refinement on *F*^2^	Primary atom site location: structure-invariant direct methods
Least-squares matrix: full	Secondary atom site location: difference Fourier map
*R*[*F*^2^ > 2σ(*F*^2^)] = 0.041	Hydrogen site location: inferred from neighbouring sites
*wR*(*F*^2^) = 0.115	H atoms treated by a mixture of independent and constrained refinement
*S* = 1.08	*w* = 1/[σ^2^(*F*_o_^2^) + (0.071*P*)^2^] where *P* = (*F*_o_^2^ + 2*F*_c_^2^)/3
7436 reflections	(Δ/σ)_max_ = 0.019
184 parameters	Δρ_max_ = 0.84 e Å^−^^3^
0 restraints	Δρ_min_ = −0.34 e Å^−^^3^

### Special details

**Table:**

Geometry. All e.s.d.'s (except the e.s.d. in the dihedral angle between two l.s. planes) are estimated using the full covariance matrix. The cell e.s.d.'s are taken into account individually in the estimation of e.s.d.'s in distances, angles and torsion angles; correlations between e.s.d.'s in cell parameters are only used when they are defined by crystal symmetry. An approximate (isotropic) treatment of cell e.s.d.'s is used for estimating e.s.d.'s involving l.s. planes.
Refinement. Refinement of *F*^2^ against ALL reflections. The weighted *R*-factor *wR* and goodness of fit *S* are based on *F*^2^, conventional *R*-factors *R* are based on *F*, with *F* set to zero for negative *F*^2^. The threshold expression of *F*^2^ > σ(*F*^2^) is used only for calculating *R*-factors(gt) *etc*. and is not relevant to the choice of reflections for refinement. *R*-factors based on *F*^2^ are statistically about twice as large as those based on *F*, and *R*- factors based on ALL data will be even larger.

### Fractional atomic coordinates and isotropic or equivalent isotropic displacement parameters (Å^2^)

**Table:**

	*x*	*y*	*z*	*U*_iso_*/*U*_eq_	
S	0.06612 (3)	0.50611 (2)	0.38496 (2)	0.01778 (8)	
P	0.03058 (3)	0.34478 (2)	0.38973 (2)	0.01212 (8)	
O1	−0.06932 (9)	0.29955 (7)	0.31509 (5)	0.01497 (17)	
C11	−0.19850 (13)	0.35845 (11)	0.28645 (7)	0.0197 (2)	
H11	−0.2199	0.4103	0.3270	0.024*	
C21	−0.30994 (14)	0.27054 (13)	0.26853 (8)	0.0265 (3)	
H21A	−0.3976	0.3074	0.2492	0.040*	
H21B	−0.2880	0.2189	0.2295	0.040*	
H21C	−0.3163	0.2285	0.3153	0.040*	
C31	−0.18259 (16)	0.42557 (13)	0.21616 (9)	0.0307 (3)	
H31A	−0.2682	0.4653	0.1965	0.046*	
H31B	−0.1084	0.4802	0.2299	0.046*	
H31C	−0.1609	0.3747	0.1766	0.046*	
Si1	0.18608 (4)	0.19371 (3)	0.30180 (2)	0.01607 (8)	
C1	0.05411 (14)	0.08448 (11)	0.26893 (8)	0.0228 (3)	
H1A	0.0714	0.0505	0.2212	0.034*	
H1B	0.0584	0.0263	0.3084	0.034*	
H1C	−0.0368	0.1191	0.2598	0.034*	
C2	0.19037 (16)	0.29925 (12)	0.22471 (8)	0.0257 (3)	
H2A	0.2019	0.2607	0.1774	0.038*	
H2B	0.1044	0.3417	0.2155	0.038*	
H2C	0.2671	0.3509	0.2406	0.038*	
C3	0.35141 (14)	0.11555 (12)	0.31640 (9)	0.0255 (3)	
H3A	0.3617	0.0785	0.2684	0.038*	
H3B	0.4272	0.1682	0.3319	0.038*	
H3C	0.3521	0.0589	0.3566	0.038*	
Si2	0.31243 (3)	0.29123 (3)	0.46123 (2)	0.01629 (8)	
C4	0.43267 (13)	0.38055 (12)	0.41764 (8)	0.0225 (3)	
H4A	0.5138	0.3973	0.4564	0.034*	
H4B	0.4602	0.3403	0.3747	0.034*	
H4C	0.3870	0.4508	0.3990	0.034*	
C5	0.38927 (14)	0.15588 (12)	0.50201 (9)	0.0253 (3)	
H5A	0.4697	0.1716	0.5415	0.038*	
H5B	0.3217	0.1142	0.5248	0.038*	
H5C	0.4166	0.1109	0.4610	0.038*	
C6	0.27586 (13)	0.37228 (13)	0.54548 (8)	0.0232 (3)	
H6A	0.3612	0.3845	0.5820	0.035*	
H6B	0.2353	0.4449	0.5281	0.035*	
H6C	0.2118	0.3299	0.5705	0.035*	
N1	0.16513 (10)	0.26205 (8)	0.38890 (6)	0.01379 (19)	
N2	−0.04109 (11)	0.31950 (8)	0.46402 (6)	0.01388 (19)	
H2	−0.0419 (15)	0.3767 (13)	0.4924 (9)	0.017*	
C7	−0.09601 (12)	0.21560 (10)	0.49410 (7)	0.0143 (2)	
C8	−0.08932 (13)	0.11580 (10)	0.44119 (7)	0.0186 (2)	
H8A	−0.1261	0.0490	0.4626	0.028*	
H8B	−0.1436	0.1320	0.3904	0.028*	
H8C	0.0060	0.1021	0.4365	0.028*	
C9	−0.24488 (13)	0.23858 (12)	0.50102 (8)	0.0228 (3)	
H9A	−0.2838	0.1715	0.5207	0.034*	
H9B	−0.2482	0.3014	0.5363	0.034*	
H9C	−0.2978	0.2576	0.4504	0.034*	
C10	−0.01281 (14)	0.18979 (11)	0.57379 (7)	0.0204 (3)	
H10A	−0.0483	0.1215	0.5940	0.031*	
H10B	0.0834	0.1786	0.5700	0.031*	
H10C	−0.0203	0.2528	0.6084	0.031*	

### Atomic displacement parameters (Å^2^)

**Table:**

	*U*^11^	*U*^22^	*U*^33^	*U*^12^	*U*^13^	*U*^23^
S	0.02695 (17)	0.01109 (14)	0.01685 (16)	−0.00040 (11)	0.00810 (12)	−0.00059 (10)
P	0.01485 (14)	0.01073 (14)	0.01114 (15)	0.00093 (10)	0.00329 (10)	−0.00069 (10)
O1	0.0162 (4)	0.0156 (4)	0.0123 (4)	0.0025 (3)	0.0005 (3)	−0.0018 (3)
C11	0.0177 (6)	0.0237 (6)	0.0160 (6)	0.0053 (5)	−0.0016 (4)	0.0004 (5)
C21	0.0183 (6)	0.0372 (8)	0.0216 (7)	−0.0017 (6)	−0.0022 (5)	0.0035 (6)
C31	0.0283 (7)	0.0317 (8)	0.0293 (8)	0.0040 (6)	−0.0019 (6)	0.0135 (6)
Si1	0.02069 (17)	0.01375 (16)	0.01521 (17)	0.00146 (12)	0.00709 (13)	−0.00205 (12)
C1	0.0300 (7)	0.0182 (6)	0.0211 (7)	−0.0017 (5)	0.0072 (5)	−0.0070 (5)
C2	0.0360 (8)	0.0242 (7)	0.0199 (7)	0.0012 (6)	0.0135 (6)	0.0019 (5)
C3	0.0258 (7)	0.0239 (7)	0.0290 (8)	0.0058 (5)	0.0105 (5)	−0.0047 (6)
Si2	0.01396 (16)	0.01862 (17)	0.01629 (18)	−0.00085 (12)	0.00281 (12)	0.00010 (13)
C4	0.0189 (6)	0.0226 (6)	0.0267 (7)	−0.0031 (5)	0.0058 (5)	0.0002 (5)
C5	0.0212 (6)	0.0270 (7)	0.0265 (7)	0.0028 (5)	0.0016 (5)	0.0071 (6)
C6	0.0183 (6)	0.0330 (7)	0.0174 (6)	−0.0049 (5)	0.0014 (5)	−0.0052 (5)
N1	0.0144 (4)	0.0143 (4)	0.0129 (5)	0.0012 (4)	0.0031 (3)	−0.0009 (4)
N2	0.0188 (5)	0.0115 (4)	0.0124 (5)	−0.0008 (4)	0.0058 (4)	−0.0021 (4)
C7	0.0165 (5)	0.0133 (5)	0.0133 (5)	−0.0009 (4)	0.0035 (4)	0.0011 (4)
C8	0.0245 (6)	0.0135 (5)	0.0181 (6)	−0.0029 (4)	0.0044 (5)	−0.0013 (5)
C9	0.0172 (6)	0.0255 (7)	0.0267 (7)	−0.0016 (5)	0.0065 (5)	−0.0007 (5)
C10	0.0275 (7)	0.0161 (6)	0.0158 (6)	0.0000 (5)	−0.0010 (5)	0.0023 (5)

### Geometric parameters (Å, º)

**Table:**

S—P	1.9578 (6)	Si2—N1	1.7954 (12)
P—O1	1.5959 (10)	Si2—C6	1.8687 (14)
P—N2	1.6356 (11)	Si2—C4	1.8694 (14)
P—N1	1.6635 (11)	Si2—C5	1.8713 (14)
O1—C11	1.4700 (15)	C4—H4A	0.9800
C11—C31	1.513 (2)	C4—H4B	0.9800
C11—C21	1.515 (2)	C4—H4C	0.9800
C11—H11	1.0000	C5—H5A	0.9800
C21—H21A	0.9800	C5—H5B	0.9800
C21—H21B	0.9800	C5—H5C	0.9800
C21—H21C	0.9800	C6—H6A	0.9800
C31—H31A	0.9800	C6—H6B	0.9800
C31—H31B	0.9800	C6—H6C	0.9800
C31—H31C	0.9800	N2—C7	1.4898 (15)
Si1—N1	1.7907 (11)	N2—H2	0.848 (16)
Si1—C2	1.8628 (14)	C7—C8	1.5228 (17)
Si1—C1	1.8637 (14)	C7—C9	1.5319 (17)
Si1—C3	1.8653 (14)	C7—C10	1.5320 (18)
C1—H1A	0.9800	C8—H8A	0.9800
C1—H1B	0.9800	C8—H8B	0.9800
C1—H1C	0.9800	C8—H8C	0.9800
C2—H2A	0.9800	C9—H9A	0.9800
C2—H2B	0.9800	C9—H9B	0.9800
C2—H2C	0.9800	C9—H9C	0.9800
C3—H3A	0.9800	C10—H10A	0.9800
C3—H3B	0.9800	C10—H10B	0.9800
C3—H3C	0.9800	C10—H10C	0.9800
			
O1—P—N2	107.98 (6)	N1—Si2—C5	109.36 (6)
O1—P—N1	99.94 (5)	C6—Si2—C5	105.15 (7)
N2—P—N1	111.55 (6)	C4—Si2—C5	113.76 (7)
O1—P—S	112.66 (4)	Si2—C4—H4A	109.5
N2—P—S	108.90 (4)	Si2—C4—H4B	109.5
N1—P—S	115.40 (4)	H4A—C4—H4B	109.5
C11—O1—P	119.84 (8)	Si2—C4—H4C	109.5
O1—C11—C31	108.69 (11)	H4A—C4—H4C	109.5
O1—C11—C21	107.59 (11)	H4B—C4—H4C	109.5
C31—C11—C21	112.01 (12)	Si2—C5—H5A	109.5
O1—C11—H11	109.5	Si2—C5—H5B	109.5
C31—C11—H11	109.5	H5A—C5—H5B	109.5
C21—C11—H11	109.5	Si2—C5—H5C	109.5
C11—C21—H21A	109.5	H5A—C5—H5C	109.5
C11—C21—H21B	109.5	H5B—C5—H5C	109.5
H21A—C21—H21B	109.5	Si2—C6—H6A	109.5
C11—C21—H21C	109.5	Si2—C6—H6B	109.5
H21A—C21—H21C	109.5	H6A—C6—H6B	109.5
H21B—C21—H21C	109.5	Si2—C6—H6C	109.5
C11—C31—H31A	109.5	H6A—C6—H6C	109.5
C11—C31—H31B	109.5	H6B—C6—H6C	109.5
H31A—C31—H31B	109.5	P—N1—Si1	119.80 (6)
C11—C31—H31C	109.5	P—N1—Si2	115.56 (6)
H31A—C31—H31C	109.5	Si1—N1—Si2	119.70 (6)
H31B—C31—H31C	109.5	C7—N2—P	133.08 (8)
N1—Si1—C2	110.35 (6)	C7—N2—H2	114.3 (11)
N1—Si1—C1	113.62 (6)	P—N2—H2	112.5 (11)
C2—Si1—C1	110.52 (7)	N2—C7—C8	111.56 (10)
N1—Si1—C3	110.20 (6)	N2—C7—C9	107.63 (10)
C2—Si1—C3	107.50 (7)	C8—C7—C9	109.89 (11)
C1—Si1—C3	104.33 (7)	N2—C7—C10	108.97 (10)
Si1—C1—H1A	109.5	C8—C7—C10	109.54 (10)
Si1—C1—H1B	109.5	C9—C7—C10	109.20 (11)
H1A—C1—H1B	109.5	C7—C8—H8A	109.5
Si1—C1—H1C	109.5	C7—C8—H8B	109.5
H1A—C1—H1C	109.5	H8A—C8—H8B	109.5
H1B—C1—H1C	109.5	C7—C8—H8C	109.5
Si1—C2—H2A	109.5	H8A—C8—H8C	109.5
Si1—C2—H2B	109.5	H8B—C8—H8C	109.5
H2A—C2—H2B	109.5	C7—C9—H9A	109.5
Si1—C2—H2C	109.5	C7—C9—H9B	109.5
H2A—C2—H2C	109.5	H9A—C9—H9B	109.5
H2B—C2—H2C	109.5	C7—C9—H9C	109.5
Si1—C3—H3A	109.5	H9A—C9—H9C	109.5
Si1—C3—H3B	109.5	H9B—C9—H9C	109.5
H3A—C3—H3B	109.5	C7—C10—H10A	109.5
Si1—C3—H3C	109.5	C7—C10—H10B	109.5
H3A—C3—H3C	109.5	H10A—C10—H10B	109.5
H3B—C3—H3C	109.5	C7—C10—H10C	109.5
N1—Si2—C6	114.73 (6)	H10A—C10—H10C	109.5
N1—Si2—C4	108.34 (6)	H10B—C10—H10C	109.5
C6—Si2—C4	105.60 (7)		

### Hydrogen-bond geometry (Å, º)

**Table:**

*D*—H···*A*	*D*—H	H···*A*	*D*···*A*	*D*—H···*A*
N2—H2···S^i^	0.848 (16)	2.631 (16)	3.4326 (13)	158.2 (14)

Symmetry code: (i) −*x*, −*y*+1, −*z*+1.
