# Supplementary figures and images for: Crystal structure of O-isopropyl [bis­(tri­methyl­sil­yl)amino](tert-butyl­amino)­phosphino­thio­ate
Source: Acta Crystallogr E Crystallogr Commun. 2015 Jan 1;71(Pt 1):o37–8. doi: 10.1107/S205698901402622X (PMC4331855; doi:10.1107/S205698901402622X)

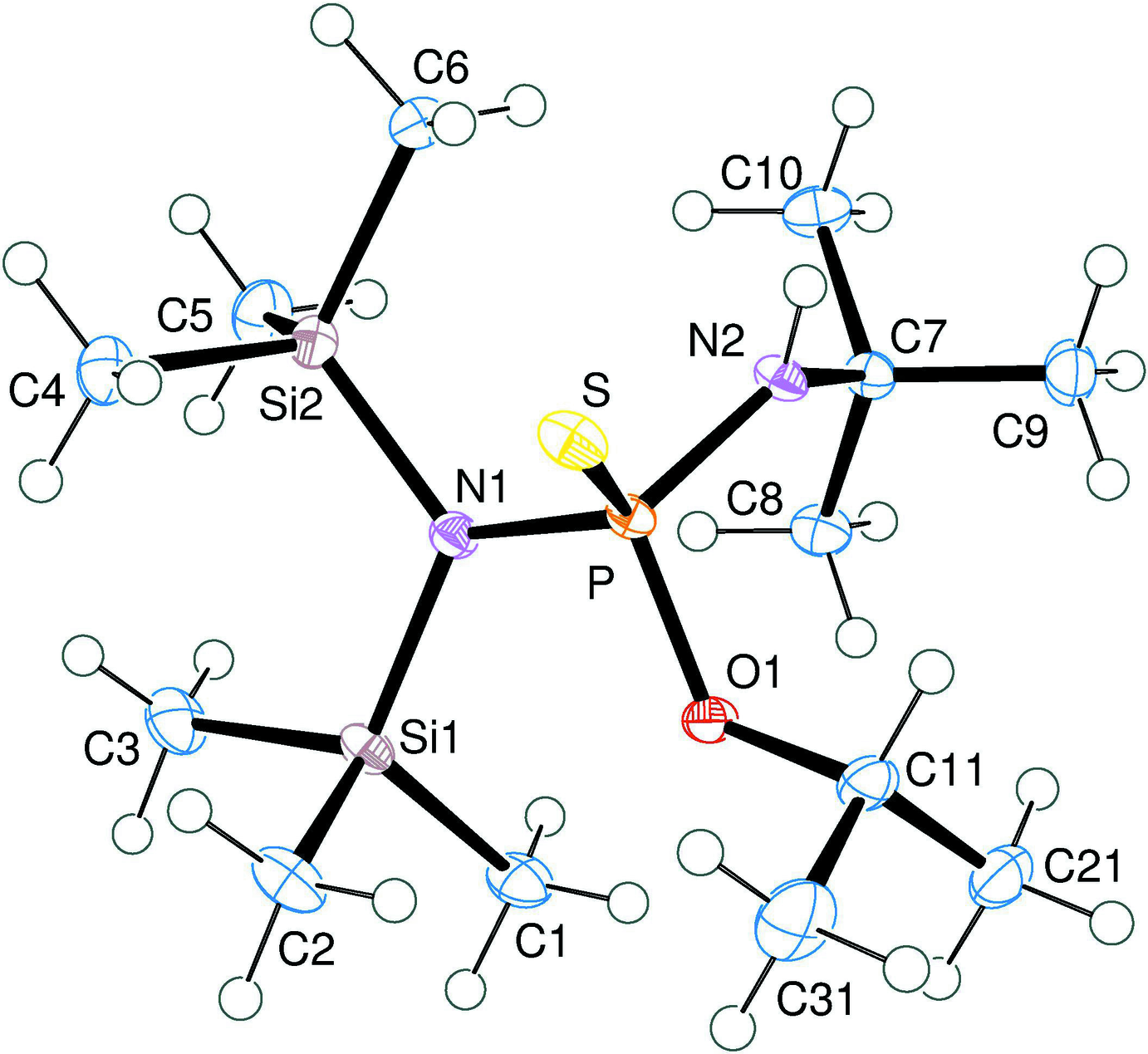

Supplement: Supplementary file 4 [file e-71-00o37-fig1.tif]
